# Effects of A Voltage Sensitive Calcium Channel Blocker and A Sodium-Calcium Exchanger Inhibitor on Apoptosis of Motor Neurons in Adult Spinal Cord Slices

**Published:** 2012-12-12

**Authors:** Hamid Reza Momeni, Mahsa Jarahzadeh

**Affiliations:** Department of Biology, Faculty of Sciences, Arak University, Arak, Iran

**Keywords:** Apoptosis, Bepridil, Loperamide, Motor Neuron, Spinal Cord

## Abstract

**Objective:**

The apoptosis of motor neurons is a critical phenomenon in spinal cord injuries. Adult spinal cord slices were used to investigate whether voltage sensitive calcium channels and Na^+^/Ca^2+^ exchangers play a role in the apoptosis of motor neurons.

**Materials and Methods:**

In this experimental research, the thoracic region of the adult mouse spinal cord was sliced using a tissue chopper and the slices were incubated in a culture medium in the presence or absence of N/L type voltage sensitive calcium channels blocker (loperamide, 100 µM) or Na^+^/Ca^2+^ exchangers inhibitor(bepridil, 20 µM) for 6 hours. 3-(4, 5-dimethylthiazol-2-yl)-2, 5 diphenyl tetrazolium (MTT) staining was used to assess slice viability while morphological features of apoptosis in motor neurons were studied using fluorescent staining.

**Results:**

After 6 hours in culture, loperamideand bepridil not only increased slice viability, but also prevented motor neuron apoptosis and significantly increased the percentage of viable motor neurons in the ventral horns of the spinal cord.

**Conclusion:**

The results of this study suggest that voltage sensitive calcium channels and Na^+^/Ca^2+^ exchanger might be involved in the apoptosis of motor neurons in adult spinal cord slices.

## Introduction

Calcium homeostasis is a tightly regulated process by which concentration of extracellular calcium is maintained at level 10000-fold higher than intracellular levels (1).

Under normal circumstances, a transient increase in the level of cytosolic calcium contributes to calcium-dependent cellular signaling. The removal of calcium from the cytoplasm is then carried out by several pumps and exchangers including calcium-ATPase pumps and Na^+^/Ca^2+^ exchangers. Undercertain pathological conditions, an extensive influx of calcium via voltage-sensitive calcium channels (2) and/or the reversed operation of Na^+^/Ca^2+^ exchangers (3) overloads the intracellular calcium concentration, triggering death signals. A number of studies have shown neuronal death induced by ischemia (4), spinal cord injuries (5), and neurodegenerative diseases (6) following elevation in the intracellular calcium concentration.When cytosolic calcium level increases, it induces necrosis signals (7). Apoptosis induced by increased intracellular calcium has also been documented (8). The application of calcium ionophores (9) and the inhibition of plasma membrane calcium pumps (10) are reports whereby apoptosis induced by elevated intracellular calcium has been shown in a wide variety of cells.

In the central nervous system, the apoptosis of motor neurons is one of the critical phenomena following spinal cord injuries (11) and neurodegenerative diseases (12) such as amyotrophic lateral sclerosis, a neurodegenerative disorder in which motor neurons in the spinal cord and motor cortex are lost. At present there is no universally accepted treatment for such diseases.

It has been shown that apoptosis could be also responsible for motor neuron death in cultured adult spinal cord slices (13, 14). However, the mechanism by which these neurons perish in culture has not yet been established. Since elevated cytosolic calcium is reported following spinal cord injuries (5) and neurodegenerative diseases (15), it could be assumed that apoptosis is induced in these neurons as a result of the uncontrolled current of calcium into the motor neurons and the resulting increased intracellular calcium levels. Based on this hypothesis, the blockage of voltage sensitive calcium channels and/or Na^+^/Ca^2+^ exchangers could be a possible way to delay apoptosis in these neurons. In accordance with this, the application of voltage sensitive calcium channel blockers (16) and Na^+^/Ca^2+^exchanger inhibitors (17) has been reported to protect neurons. The present study was thus designed to investigate the role of both a voltage sensitive calcium channel blocker and a Na^+^/Ca^2+^ exchanger inhibitor on the apoptosis of motor neurons in adult mouse spinal cord slices.

## Materials and Methods

### Preparation of organotypic spinal cord slices and treatments

This experimental study was approved by the Ethical Committee of Arak University. Adult female Balb/c mice (23-25 g) were purchased from the Pasteur Institute, Tehran, Iran. The animals were housed in plastic cages at 20℃, under a 12-hour light/dark cycle, and fed with standard commercial laboratory chew and water. The animals were deeply anesthetized by an intraperitoneal injection of sodium pentobarbital (60 mg/kg) and subsequently killed by heart puncture. The spinal cord was dissected and placed in ice cold phosphate buffered saline (PBS), pH=7.4. The thoracic region of the spinal cord was then sliced transversally into 400 µm-thick sections using a McIlwain tissue chopper (Stoelting, USA). The slices were divided into four groups: 1. Freshly prepared slices (0 hour), 2. Control slices which were cultured for 6 hours in medium, 3. Slices treated with loperamide hydrochloride (N/L type voltage sensitive calcium channels blocker, Sigma, USA, 100 µM) for 6 hours 4. Slices treated with bepridil hydrochloride (Na^+^/Ca^2+^ exchanger inhibitor, Sigma, USA, 20 µM) for 6 hours. Loperamide and bepridil were prepared as stock solutions in dimethylsulfoxide (DMSO) and stored in aliquots at -20℃. Aliquots of the stock solution were directly added to the medium. The controls received a corresponding amount of DMSO. The control and the treated slices were then placed in a four-well sterile plastic plate where each well contained 450 µl medium composed of a mixture of 50% minimum essential medium, 25% Hanks balanced salt solution, 25% horse serum, 25 mMN-2-hydroxyethyl piperazine-N’-2-ethanesulfonic acid (HEPES), 6 g/L glucose and 1% penicillin-streptomycin, pH=7.3-7.4). The cultures were incubated at 37℃ in a humidified atmosphere of 5% CO_2_ in air.

### Fixation and sectioning

The slices were fixed in Stefanini’s fixative (2% paraformaldehyde, 0.2% picric acid in 0.1 M phosphate buffer, pH=7.2) for at least 2 hours. The fixed slices were washed in PBS (3×5 minutes) and incubated overnight in 20% sucrose in PBS at 4℃. The slices were cut into 10 µm-thick sections using a cryostat (Leica, Germany). The sections were collected and mounted on Poly-L-lysine coated glass slides.

### 3-(4, 5-dimethylthiazol-2-yl)-2,5-diphenyltetrazolium bromide staining

The MTT (Sigma, USA) staining was used to assess the slice viability. MTT was dissolved in PBS as a 5mg/ml stock solution. The freshly prepared slices and the cultured slices (control and treatment groups) were transferred into four-well sterile plastic plates where each well contained 450 µl the culture medium (four slices in each well). For assay, 50 µl of the stock solution was added to the culture medium and incubated at 37℃ for 20 minutes. The slices were then photographed using a bright field microscope.

### Assessment of apoptosis

To study morphological features of apoptosis, the combination of propidium iodide (PI, Sigma, USA, 10 µg/ml in PBS, 15 minutes at room temperature) and Hoechst 33342 (Sigma, USA, 10 µg/ml in PBS, 1 minute at room temperature) was used. The cryostat sections were washed in PBS (3×5 minutes), mounted in glycerol/PBS (1:1) and coverslipped. Digital photographs were taken with an Olympus camera attached to an Olympus fluorescence microscope (Olympus Optical Co Ltd, Japan) using the appropriate excitation and emission filters.

### Motor neuron count

The percentage of viable motor neurons was estimated by counting 12 randomly selected ventral horns from each experiment.

### Statistical analysis

Results were expressed as mean ± SD. The statistical significances were analyzed by analysis of variance (ANOVA). In all cases, p<0.05 was considered significant

## Results

### Loperamide and bepridil increased slice viability

**Fig 1:**
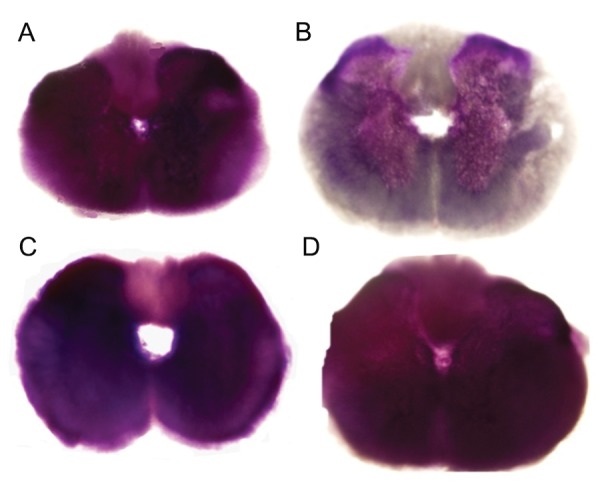
MTT staining for assessing spinal cord slice viability. A. Freshly prepared slice (0 hour). B. Slice cultured for 6 hours (control). The viability of slices cultured for 6 hours in the presence of N/L typevoltage sensitive calcium channel blocker (loperamide hydrochloride, 100 µM),C. orNa^+^/Ca^2+^ exchanger inhibitor (bepridil hydrochloride, 20 µM), D. was considerably increased. Magnification: ×40.

In freshly prepared slices (0 hour), intense MTT staining was observed in both white and gray matter (Fig 1A). In slices cultured for 6 hours (control), the staining in the white matter and the ventral horns were less pronounced (Fig 1B). The application of N/L type voltage sensitive calcium channel blocker (loperamide hydrochloride, 100 µM) (Fig 1C) and Na^+^/Ca^2+^ exchanger inhibitor (bepridil hydrochloride, 20 µM) (Fig 1D) in culture, considerably increased the slice viability after 6 hours compared to the control.

### Inhibition of apoptosis in motor neurons by loperamide and bepridil

**Fig 2:**
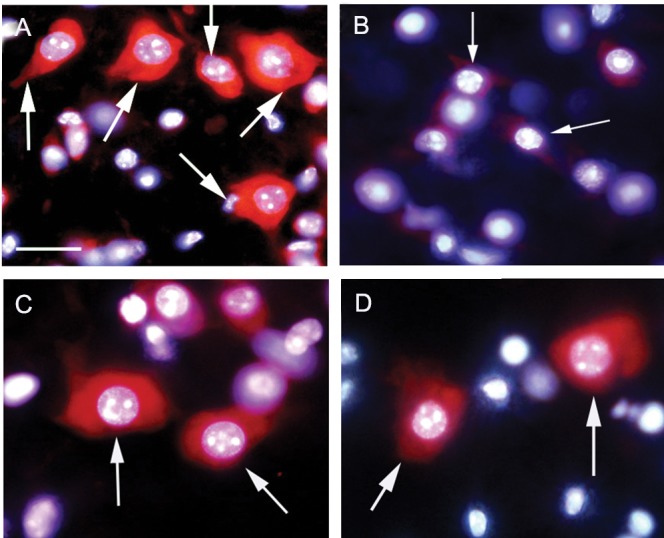
The effect of N/L type voltage sensitive calcium channel blocker (loperamide hydrochloride) and Na^+^/Ca^2+^ exchanger inhibitor (bepridil hydrochloride) on the apoptosis of motor neurons. A. Normal motor neurons from freshly prepared slices (0 hour). B. Motor neurons from slices cultured for 6 hours (control) displayed morphological features of apoptosis. The application of loperamide hydrochloride, 100 µM, C. or bepridil hydrochloride, 20 µM, D. could prevent apoptosis in the motor neurons from slices cultured for 6 hours. Arrows point out motor neurons. Scale bar: 20 µm.

**Fig 3:**
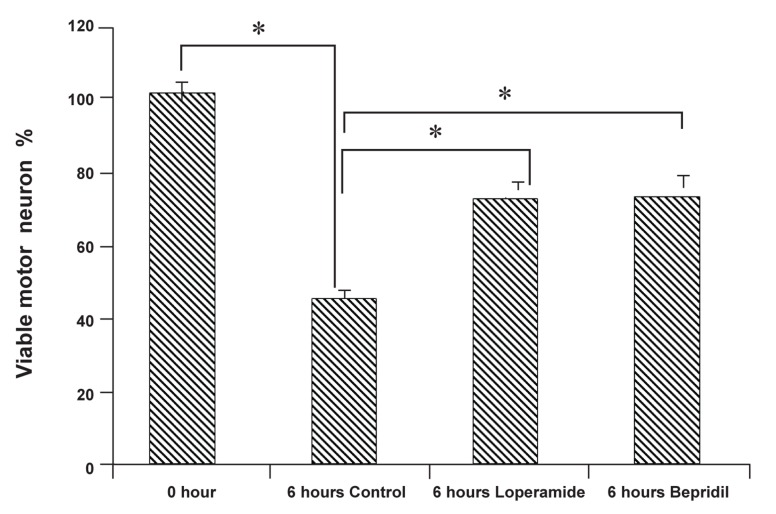
The effect of N/L type voltage sensitive calcium channel blocker (loperamide hydrochloride) and Na^+^/Ca^2+^ exchanger inhibitor (bepridil hydrochloride) on motor neuron viability. The percentage of viable motor neurons was significantly increased in slices exposed to loperamide hydrochloride, 100 µM, or bepridil hydrochloride, 20 µM, after 6 hours. Mean ± SD, n=12. *p<0.01.

In the spinal cord sections, the motor neurons were identified by morphological characteristics (large cell bodies and large nuclei) and their location (ventral horns). In freshly excised slices (0 hour), the motor neurons appeared intact with large cell bodies, large nuclei and the expected distribution of nuclear chromatin without any apoptotic signs (Fig 2A). In contrast, motor neurons cultured for 6 hours (control) displayed morphological apoptotic changes including nuclear and chromatin condensation (Fig 2B) as compared with the motor neurons at 0 hour. The application of N/L type voltage sensitive calcium channel blocker (loperamide hydrochloride, 100 µM) (Fig 2C) or Na^+^/Ca^2+^ exchanger inhibitor (bepridil hydrochloride, 20 µM) (Fig 2D) for 6 hours effectively inhibited nuclear and chromatin condensation in the motor neurons. In addition, loperamide and bepridil significantly increased the percentage of viable motor neurons in the ventral horns after 6 hours as compared with the control (Fig 3).

## Discussion

In the present study, adult spinal cord slices were used to investigate one of the possible mechanisms involved in the apoptosis of motor neurons in cultured spinal cord slices. Results showed that N/L type voltage sensitive calcium channel blocker as well as inhibition of the Na^+^/Ca^2+^ exchanger not only increased the viability of the cultured slices but also inhibited apoptosis in the motor neurons and significantly increased the percentage of viable motor neurons in the ventral horns.

The MTT assay was used to evaluate qualitative viability in the spinal cord slices. This method, which results in the formation of purple formazan, is widely used to assess cell survival and proliferation (18, 19). The MTT method measures mitochondrial integrity but it should be born in mind that MTT reduction can also be caused by dehydrogenases outside the mitochondria (20). However, the MTT assay is still a convenient method for assessing cell viability in brain and spinal cord slices and it can also be used to localize the areas of cell death within the slices (21).

In the present study and in our previous results (13, 14), we have shown that motor neurons in cultured spinal cord slices display both morphological and biochemical features of apoptosis after 6 hours. Controversy remains regarding the mechanisms by which apoptosis is induced in the motor neurons. Based on the hypothesis of calcium-mediated apoptosis (8) and neural death induced by intracellular calcium overload following spinal cord injury (5), it is reasonable to assume that apoptosis induced in the motor neurons of cultured spinal cord slices might be due to an elevation in the cytosolic calcium of theses neurons. If this hypothesis is true, the prevention of excessive calcium influx should inhibit apoptosis in the motor neurons. N and L type voltage sensitive calcium channels are found in spinal cord neurons (22). These channels are one of ways by which calcium enters the neurons and the channels play an important role in apoptosispathways under pathological conditions (23, 24). The present results showed that loperamide (N/L type voltage sensitive calcium channel blocker) not only increased the viability of the cultured slices but also inhibited apoptosis in the motor neurons and increased the number of viable motor neurons in the ventral horns. It is therefore likely that in the cultured slices some sort of stimuli induced a voltage change in the motor neurons, leading to an excessive influx of calcium and therefore an increase in the intracellular calcium concentration.

Under pathological conditions, another mechanism likely to result in extensive calcium influx is the reverse operation of Na^+^/Ca^2+^ exchangers, resulting in an overload in the intracellular calcium level (3). It could be hypothesized that at early time point of slice culture, some factors, such as the dysfunction of Na^+^-K+ ATPase, the Na^+^/Ca^2+^ exchanger could have reversed, leading to increased cytosolic calcium levels in the motor neurons. To test this hypothesis, bepridil hydrochloride (Na^+^/Ca^2+^ exchanger inhibitor) was used.The application of this inhibitor increased the viability of the cultured slices. In addition, it inhibited apoptosis in the motor neurons as well as increased the number of viable motor neurons in the ventral horns.

Taken together, these results suggest that the mechanisms underlying elevated intracellular calcium concentrations in the motor neurons may be due to voltage sensitive calcium channels and/or Na^+^/Ca^2+^ exchangers. However, the signaling pathway by which elevated intracellular calcium concentrations induce apoptosis in motor neurons remains to be investigated. A logical relationship could be the activation of calcium-dependent proteases and endonucleases. In addition, transient increases in cytosolic calcium have been shown to stimulate the mitochondrial uptake of calcium (25). The accumulation of mitochondrial calcium leads to a change in mitochondrial voltage and the opening of the permeability transition pore (PTP). This in turn results in the release of apoptogenic proteins including apoptosis-inducing factor (AIF), endonuclease G and cytocrome c from the mitochondria (25, 26). It should be born in mind that the excessive influx of calcium can also occur following over-activation of calcium permeable glutamate receptors (27). The application of glutamate receptor antagonists in the cultured spinal cord slices is therefore suggested in further investigations.

## Conclusion

Our results suggest a key role for calcium in triggering apoptosis in the motor neurons of cultured spinal cord slices. They also show that voltage sensitive calcium channels blocker andthe inhibition of Na^+^/Ca^2+^ exchangers delay apoptosis in these neurons.
